# Expression of Hypoxia-Inducible Factor 1a (HIF-1a), Regulatory T Cells (Treg) and T Helper 17 Cells (Th17) in PCOS Phenotype D Patients from Polish Population

**DOI:** 10.3390/ijms27073108

**Published:** 2026-03-29

**Authors:** J. Kuliczkowska-Płaksej, D. Szymczak, J. Halupczok-Żyła, M. Strzelec, A. Podsiadły, N. Słoka, M. Bolanowski, B. Stachowska, A. Zdrojowy-Wełna, A. Jawiarczyk-Przybyłowska

**Affiliations:** 1 Department of Endocrinology and Internal Diseases, Wroclaw Medical University, 50-367 Wroclaw, Poland; 2 Department and Clinic of Hematology, Blood Neoplasms, and Bone Marrow Transplantation, Wroclaw Medical University, 50-367 Wroclaw, Poland; 3 Department of Clinical and Experimental Pathology, Wroclaw Medical University, 50-367 Wroclaw, Poland

**Keywords:** polycystic ovary syndrome, low-grade inflammation, Treg, Th17, HIF-1α

## Abstract

Polycystic ovary syndrome (PCOS) is associated with reproductive, metabolic, and inflammatory disturbances. Alterations in T-cell subpopulations—particularly increased T helper 17 cells (Th17) and decreased regulatory T cells (Treg)—have been reported in PCOS; however, data on normoandrogenic phenotype D remain limited. Hypoxia-inducible factor 1α (HIF-1α), a key regulator of hypoxic response, also influences immune and metabolic processes and may affect the Treg/Th17 balance. To assess Treg and Th17 abundance, HIF-1α expression within these cells, and their ratios in women with phenotype D PCOS compared with healthy controls. The study included 49 women with phenotype D PCOS and 40 controls comparable in terms of age and BMI. Anthropometric, hormonal, metabolic, and inflammatory parameters were evaluated. Peripheral T-cell subsets and intracellular HIF-1α expression were analyzed by multiparameter flow cytometry. Absolute numbers of Treg and Th17 cells did not differ between groups. However, PCOS patients showed significantly higher Treg/Th17 and HIF-1α-positive Treg/HIF-1α-positive Th17 ratios. HIF-1α-positive Treg cells correlated positively with adiposity and insulin resistance markers; however, after False Discovery Rate (FDR) correction, correlations no longer remained statistically significant. Despite normoandrogenemia, PCOS patients exhibited higher hs-CRP levels. Phenotype D PCOS is characterized by altered immune cell ratios rather than absolute T-cell differences, suggesting distinct immunological features and persistent low-grade inflammation.

## 1. Introduction

Polycystic ovary syndrome (PCOS) is one of the most common endocrinopathies among women of reproductive age, affecting as many as 5–25% of this population [1]. PCOS is a heterogeneous disorder with complex and multifactorial etiologies, resulting in a wide range of clinical and biochemical manifestations. To reflect this variability, PCOS has been classified into four main phenotypes. Phenotype D, also known as normoandrogenic PCOS, was introduced in the 2003 Rotterdam diagnostic criteria [2], which remain the most widely accepted diagnostic standard. This phenotype is characterized by normal androgen levels, relatively mild endocrine disturbances, and a lower prevalence of metabolic complications compared with other PCOS phenotypes [3,4,5]. Accordingly, affected individuals typically do not exhibit clinical signs of hyperandrogenism, such as hirsutism, and usually present with a normal modified Ferriman–Gallwey score [3,6]. In contrast, phenotypes A, B, and C are associated with hyperandrogenism and a higher risk of metabolic dysfunction [4,7]. The ongoing debate regarding the diagnostic relevance of phenotype D highlights the heterogeneity of PCOS and suggests potential differences in underlying pathophysiology between normoandrogenic and hyperandrogenic phenotypes [8,9,10,11]. In addition to menstrual and fertility disorders, PCOS is associated with an increased risk of cardiovascular and metabolic complications [1]. Many studies indicate a significant role of low-grade inflammation in the pathogenesis of PCOS, with the syndrome itself also recognized as a pro-inflammatory state [12,13,14,15]. Patients with PCOS have been found to exhibit higher concentrations of pro-inflammatory cytokines, as well as elevated leukocyte counts and a higher percentage of neutrophils in peripheral blood [16,17]. Some studies suggest an imbalance in T lymphocytes in women with PCOS—characterized by higher numbers of Th1 lymphocytes and lower numbers Th2 lymphocytes [16,18,19]. This may indicate dysregulation of the immune system. Inflammation, including leukocytosis, is associated with the later development of metabolic disorders such as obesity and insulin resistance (IR), which is a key component in the etiology of PCOS [16,18]. Hypoxia-inducible factors (HIFs) are transcription factors that regulate the cellular response to hypoxia. Hypoxia-inducible factor 1α (HIF-1α) is detected in almost all cell populations, including macrophages, dendritic cells, neutrophils, and lymphocytes [20,21,22]. Beyond its primary role in regulating the immune response, HIF-1α also participates in several other processes, including glucose, fatty acid, and cholesterol metabolism [20,21,22,23]. Dysregulation of HIF-1α is associated with the development of obesity, nonalcoholic fatty liver disease, and type 2 diabetes [24,25,26]. Excessive activation of HIF-1α has been demonstrated in the adipose tissue of obese individuals, leading to increased local inflammation and IR [24,25,26]. HIF-1α expression is activated not only by hypoxia but also through the effects of hyperglycemia, inflammatory proteins, and insulin [20,21,23,24,27]. The results of some studies suggest that activation of HIF-1α directly regulates T-cell differentiation and may selectively increase the number of regulatory T cells (Tregs), which are responsible for maintaining self-tolerance and play a role in preventing early miscarriages [28]. However, studies on the effect of HIF-1α activation on Treg numbers are inconclusive: many reports suggest that increased HIF-1α activation in lymphocytes leads to reduced Treg counts and an increase in pro-inflammatory T helper 17 cells (Th17) [29,30,31]. Results of studies on the abundance of T-cell subpopulations in PCOS patients are also inconsistent [32,33]. Some studies indicate an increased number of pro-inflammatory Th17 lymphocytes and a decreased number of Treg cells in patients with PCOS compared with healthy, fertile women, which may contribute to the obstetric complications typically observed in PCOS [34]. The relative dominance of these populations can mean the difference between autoimmune pathology and tolerance. In addition, because HIF-1α responds to metabolic cues, this provides a basis for understanding how the metabolic environment can affect T-cell differentiation. Therefore, a clear understanding of the role of metabolism in shifting this balance and identifying new modifiable targets is directly relevant to many human diseases, including PCOS.

The aim of our study was to assess the numbers of Th17 and Treg lymphocytes in a population of patients with phenotype D PCOS compared to healthy women with regular menstrual cycles. Additionally, we evaluated HIF-1α expression in both T-cell subpopulations, as well as the Treg/Th17 ratio and the ratio of both lymphocyte types expressing HIF-1α.

## 2. Results

No statistically significant differences were found between the groups with respect to body weight or BMI. However, both waist and hip circumferences were significantly greater in women with PCOS compared with controls, while WHR did not differ between the groups. In terms of hormonal profile, women with PCOS exhibited significantly higher serum concentrations of testosterone and LH, as well as elevated FAI values, although androgen levels and FAI generally remained within the normal reference range. In addition, hs-CRP concentrations were significantly higher in the PCOS group. The baseline characteristics of the PCOS and control group are summarized in Table 1.

Hormonal and biochemical profiles in women with PCOS and the control group are presented in Table 2.

No significant differences were observed in the numbers of leukocytes or lymphocytes, including T lymphocytes, CD4+ T lymphocytes, Treg cells, and Th17 cells, between patients with PCOS and the control group. Analysis of HIF-1α-positive lymphocyte subpopulations revealed no statistically significant differences between the two groups in the numbers of Th17 cells, Treg cells, CD3+ T lymphocytes, or CD4+ T lymphocytes expressing HIF-1α. However, the Treg/Th17 ratio and the Treg HIF-1α+/Th17 HIF-1α+ ratio were significantly higher in the PCOS group compared with the control group (the statistical power of the Mann–Whitney test was, respectively, 0.7705 and 0.5615). Detailed peripheral blood count parameters in both groups are presented in Table 3.

In the PCOS group, statistically significant but weak positive correlations were observed between the number of Treg cells and several clinical parameters, including BMI values (r = 0.359), waist (r = 0.392) and hip circumferences (r = 0.392), testosterone concentration (r = 0.28), and FAI values (r = 0.34). HIF-1α-positive Treg cells showed positive correlations with insulin levels (r = 0.32), HOMA-IR (r = 0.301), total cholesterol (r = 0.337), BMI (r = 0.323), and waist (r = 0.32) and hip circumferences (r = 0.303). HIF-1α-positive Th17 cells were negatively correlated with LH levels (r = −0.31). In the control group, statistically significant correlations were observed only within the HIF-1α-positive Treg and Th17 cell subgroups. HIF-1α-positive Treg cells correlated positively with BMI values (r = 0.37), waist (r = 0.42) and hip circumferences (r = 0.36), insulin levels (r = 0.453), and HOMA-IR values (r = 0.42), whereas HIF-1α-positive Th17 cells showed positive correlations with insulin levels and HOMA-IR (r = 0.48). However, these associations did not remain statistically significant after FDR correction.

## 3. Discussion

In our study, we evaluated patients with phenotype D PCOS, which is characterized by menstrual disorders, polycystic ovarian morphology on ultrasound, and normoandrogenemia. Patients with phenotype D PCOS are characterized by a lower severity of metabolic and hormonal disturbances compared with the classical variants of this syndrome [3,4,5]. Patients from this group in our study, despite normoandrogenemia, differed significantly in testosterone concentrations as well as FAI values compared with the control group. We did not observe significant differences in metabolic and anthropometric parameters, despite significantly higher waist and hip circumferences in the PCOS group. The main aim of our study was to assess the numbers of Treg cells and Th17 cells in the PCOS group in comparison with the control group. The pathogenesis of PCOS is complex and multifactorial. Increasing evidence suggests that beyond hormonal disturbances, immune imbalance may substantially contribute to its development [17,35,36]. Previous studies have shown that women with PCOS exhibit not only elevated white blood cell counts and chronic low-grade inflammation but also increased levels of autoantibodies, including anti-nuclear, anti-thyroid, and anti-islet cell antibodies [12,37]. Additionally, PCOS has been associated with a three-fold higher risk of recurrent miscarriage [38]. In this context, the importance of distinct T lymphocyte subsets, particularly T helper (Th) subsets, in the regulation of the immune response has been demonstrated [39,40,41,42,43,44,45,46]. In recent years, attention has focused mainly on Th17 and Treg cells. Tregs exert anti-inflammatory effects by suppressing T-cell proliferation and autoimmune responses [47,48]. Tregs are also essential for maternal–fetal tolerance, implantation, and pregnancy maintenance [40,42,43]. Reduced Treg proportions have been reported in pregnancy complications such as recurrent miscarriage and preeclampsia [40,42,43]. In contrast, Th17 cells are involved in host defense against extracellular pathogens but may also promote autoimmune and inflammatory conditions, including pregnancy-related pathologies [45,46,49,50,51,52]. Notably, both subsets arise from the same precursor CD4+ T cells upon stimulation by an antigen [53]. Patients in the PCOS group in our study did not differ significantly in leukocyte or lymphocyte counts compared with the control group; however, they were characterized by significantly higher hs-CRP concentrations. PCOS is a syndrome associated with low-grade inflammation, which additionally contributes to the exacerbation of metabolic disturbances [12,13,14,15,54]. In our study, we did not find a statistically significant difference in the numbers of Treg and Th17 lymphocytes between the two groups. Additionally, we evaluated the Treg/Th17 ratio between the two study groups. We observed a significant difference in the ratio, and surprisingly, the higher ratio was observed in patients with PCOS, indicating a predominance of Treg cells in the population of patients with this syndrome. This observation is inconsistent with the results of previous studies on Treg and Th17 cell abundance in patients with PCOS. Nasri et al. examined the distribution of Th1/Th2/Th17/Treg subsets in the peripheral blood of infertile women diagnosed with PCOS and compared the findings with those from healthy fertile controls. They reported a higher proportion of Th17 cells in women with PCOS; however, this increase did not reach statistical significance. In contrast, evaluation of the Th17/Th2 ratio revealed a significant shift toward Th17 predominance in the PCOS group [55]. The authors also identified a markedly lower percentage of Treg cells in women with PCOS compared with healthy controls. Although a negative association between Th1 and Treg subsets was observed in the PCOS group, this correlation was not statistically significant. Furthermore, they assessed both Th1/Treg and Th17/Treg ratios and concluded that the Th17/Treg balance appears to be particularly relevant in the immunological context of PCOS. Based on these findings, the authors suggested that, in addition to Th1/Th2 and Treg cells, Th17 cells contribute substantially to the immune mechanisms underlying PCOS. The reduction in Treg numbers in PCOS was further corroborated by findings reported by Wu et al. [56]. Krishna et al. additionally demonstrated that impaired interleukin-2-Signal Transducer and Activator of Transcription 5B-Forkhead box P3 (IL2-STAT5B-FOXP3) signaling may underlie decreased Treg levels in women with PCOS [33]. Earlier research showed that in healthy fertile women, Treg numbers increase progressively during the follicular phase of the menstrual cycle in parallel with rising estrogen concentrations [57]. The increase in Tregs during the late follicular phase is considered crucial for establishing the immune tolerance required for successful embryo implantation [57]. In contrast, women with PCOS appear to exhibit defective Treg expansion during this phase [33,57]. Estradiol has been identified as a key regulator of peripheral Treg differentiation and suppressive activity, while testosterone may also directly or indirectly contribute to maintaining Treg homeostasis, particularly in males [58,59].

PCOS is characterized by endocrine abnormalities, most notably hyperandrogenemia. In the study by Krishna et al., women with PCOS exhibited elevated testosterone levels together with reduced Treg counts, a finding that was difficult to explain mechanistically. The authors proposed that women with PCOS may possess an intrinsic defect in peripheral Treg induction despite increased androgen levels. Their correlation analysis revealed no significant associations between Treg percentages and most hormonal or physiological parameters, including estradiol and testosterone; only LH showed a significant correlation with Treg levels in healthy women [33]. In our study, although in the initial analysis we observed a significant, albeit weak, positive correlation between Treg cell numbers, and FAI and testosterone in women with PCOS, it did not remain statistically significant after FDR correction for multiple testing. This may reflect the moderate sample size and the relatively large number of analyzed parameters. Therefore, these findings should be interpreted as exploratory. Considering the results of previous studies suggesting a positive effect of androgens on Treg cell numbers, it might be expected that in the case of PCOS phenotype D, significantly higher testosterone concentrations (although still within the normal range) compared with an age-, weight-, and BMI-matched population of healthy women exert a protective effect against a reduction in Treg numbers. Nevertheless, this hypothesis requires validation in studies including larger patient populations.

Another parameter assessed was the expression of HIF-1α in both T-cell populations, as well as in CD3+ T lymphocytes and CD4+ helper T lymphocytes. We did not reveal statistically significant differences between the two groups in the number of T lymphocytes expressing HIF-1α. We also assessed the ratio of HIF-1α-expressing Treg lymphocytes to HIF-1α-expressing Th17 lymphocytes, which was significantly higher in PCOS patients in comparison with the control group.

HIFs are transcription factors that control the expression of genes activated under low-oxygen conditions, thereby enabling cellular adaptation to hypoxia [20,23,60,61]. The HIF complex is a heterodimer consisting of an oxygen-sensitive α-subunit and a constitutively expressed β-subunit. HIF-1α is widely expressed across tissues, whereas HIF-2α shows more restricted, tissue-specific expression [20,21,22,23,62]. Under hypoxic conditions, HIF activates numerous genes involved in angiogenesis, cell proliferation, migration, autophagy, and apoptosis [27,63,64]. Hypoxia plays a significant role in the pathophysiology of several disorders, including obesity, IR, and diabetes, all of which are characterized by chronic inflammation [65,66,67]. Importantly, HIF-1α may also be regulated independently of oxygen availability, for example in response to hyperglycemia, oxidative stress, or inflammatory mediators [63,64,68]. Dysregulated HIF signaling has been implicated in obesity, fatty liver disease, and type 2 diabetes, where it contributes to inflammation and IR [65,66]. The influence of HIF-1α on T lymphocyte differentiation, function, and abundance remains incompletely understood. Early studies suggested that HIF-1α suppresses T-cell activation and proliferation [69,70,71]. Disrupting HIF-1α in T cells results in decreased expression of several glycolysis genes [28,30,72,73]. These observations led to the hypothesis that hypoxia and HIF-1α may serve to restrain T-cell-driven inflammation, a concept that was first tested by Sitkovsky [74]. HIF-1α was also shown to regulate the Th17/Treg balance. Some studies indicate that HIF-1α promotes both Treg and Th17 differentiation [29,30]. For example, Clambey et al. presented data indicating that FOXP3 is a direct HIF-1α target gene, and hypoxia has been shown to enhance Treg abundance through transcriptional activation of FOXP3 mRNA by HIF-1α and hypoxia-driven transforming growth factor β (TGF-β)-dependent mechanisms [75]. They also demonstrated that HIF-1α is required for optimal suppressive function of Tregs [75]. The observation that hypoxia, via HIF-1α, promotes Tregs is consistent with a previous report by Ben-Shoshan et al. [76]. The authors showed that hypoxia increased FOXP3 expression in mouse splenocytes and human peripheral blood mononuclear cells [76]. In contrast to these findings, other investigations indicate that HIF-1α is crucial for the development of pro-inflammatory Th17 cells while simultaneously restricting Treg differentiation [29,30]. This interpretation stems from observations that genetic deletion of HIF-1α impairs Th17 differentiation and, at the same time, promotes the generation of Tregs [29]. Shi et al. demonstrated that Th17 cells express particularly high levels of HIF-1α, supporting the concept that this factor plays a central role in driving Th17 lineage commitment [30]. Dang et al. further explored this mechanism and showed that HIF-1α modulates the Th17/Treg balance by inducing retinoic acid-related orphan receptor γt (RORγt), thereby activating Th17-associated genes, while concurrently limiting FOXP3 protein levels through HIF-1α-dependent protein degradation [29]. In agreement with these findings, Clambey et al. found that culturing T cells in Th17-inducing conditions in hypoxia enhances Th17 cell generation [75]. However, when T cells were cultured under Treg-inducing culture conditions, hypoxia did not promote Th17 differentiation but instead promoted Treg differentiation. Moreover, in contrast to Dang et al., the authors did not observe reduced FOXP3 protein in Tregs cultured in hypoxia [75]. It is also noteworthy that elevated HIF-1α expression is not exclusive to Th17 cells; inducible Tregs likewise exhibit higher HIF-1α levels than naive CD4+ T cells [29]. Previous research has shown that hypoxia stimulates TGF-β production, which supports differentiation of both Tregs and Th17 cells [53,77]. Conversely, numerous studies suggest that HIF-1α actively suppresses Treg differentiation by targeting FOXP3 for proteasomal degradation [29,71]. HIF-1α may weaken Treg development and function through multiple mechanisms, including FOXP3 degradation, destabilization of Tregs under both normoxic and hypoxic conditions, and metabolic reprogramming toward glycolysis that favors Th17 differentiation at the expense of Tregs [29]. Overall, the net effect of hypoxia on T-cell differentiation likely reflects a complex interaction among HIF-1α, TGF-β, and the surrounding cytokine environment.

In our study, HIF-1α-positive Treg cells in women with PCOS were weakly but significantly positively correlated with several metabolic parameters, but after FDR correction for multiple testing, they did not remain statistically significant.

Data concerning HIF-1 expression and alterations in the HIF-1 pathway in PCOS remain inconsistent. Most studies investigating HIF-1α expression in patients with PCOS have focused on its expression in follicular fluid and ovarian follicular cells. Moreover, the majority of available studies have been conducted using animal models of PCOS. Studies concerning HIF-1α expression within granulosa cells have demonstrated that HIF-1a expression increases in developing follicles and that its upregulation stimulates glucose uptake. Conversely, reduced HIF-1α mRNA and protein expression has been documented in granulosa cells from women with PCOS and in ovaries of PCOS-like rats [28,78,79,80]. Impaired expression of HIF-1α in granulosa cells from PCOS patients, possibly related to mitochondrial dysfunction and aberrant HIF-1α signaling pathways, has been implicated in abnormal folliculogenesis in PCOS [78,81]. Expression of HIF-1α has also been assessed within endometrial tissue [82]. Reduced HIF-1α mRNA and protein expression has also been reported in endometrial tissue from women with PCOS. The authors hypothesized that HIF-1α may be involved in the molecular mechanisms of endometrial dysfunction in women with PCOS [82]. In contrast, other studies have shown increased levels of HIF-1α mRNA and protein induced by reactive oxygen species [78,82,83].

Padmanabhan et al. discovered decreased expression of the transcription factor Autoimmune Regulator (AIRE) in peripheral blood mononuclear cells of women with PCOS, which contributes to increased HIF-1α and reduced FOXP3 [84]. In healthy individuals, AIRE suppresses HIF-1α transcription, thereby protecting FOXP3 from degradation, while in PCOS patients, reduced AIRE expression may fail to inhibit HIF-1α transcription [84]. The results of our study indicate that both the Treg/Th17 ratio and the ratio of HIF-1α-expressing Treg cells to HIF-1α-expressing Th17 cells are elevated in women with PCOS phenotype D, findings that are difficult to explain unequivocally. A clear interpretation of the obtained results is difficult due to the lack of data on the abundance of these lymphocyte populations, as well as on HIF-1α expression in this PCOS phenotype. This issue, along with the immunological profile of patients with phenotype D PCOS, has not been investigated to date. The pattern obtained in our study may suggest that HIF-1α expression is increased in the Treg population of patients with PCOS. It is possible that in the PCOS D phenotype, characterized by the least severe hormonal and metabolic disturbances, the number of Treg cells does not decrease (as observed in the classic phenotypes), but instead there is an increased expression of HIF-1α, reflecting ongoing inflammation. However, the functional implications of elevated HIF-1α in Tregs are uncertain. A higher ratio of HIF-1α-expressing Treg lymphocytes to HIF-1α-expressing Th17 cells is not unequivocally beneficial; it is possible that increased HIF-1α expression within Treg cells themselves exerts an unfavorable effect on the function and viability of this lymphocyte population. On the other hand, considering the results of previous studies indicating the dual effect of HIF-1α on Treg function—on the one hand reducing their expression, and on the other hand exerting a positive effect by enhancing the anti-inflammatory activity of Treg cells—it is difficult to assess the impact of the increased number of HIF-1α-expressing Treg cells observed in our study compared with the control group. An increase in HIF-1α expression within Th17 or Treg lymphocyte populations does not necessarily reflect its functional activity and does not allow us to determine its impact on lymphocyte function with complete certainty.

The conclusion of our study is that in patients with the PCOS D phenotype, the numbers of Treg and Th17 cells did not differ compared with the control group; however, the Treg/Th17 ratio and the ratio of HIF-1α-expressing Treg cells to HIF-1α-expressing Th17 cells were significantly higher in the PCOS group than in the control group. This may reflect low-grade inflammation in patients with PCOS; however, the exact significance of the increased HIF-1α expression within Treg cells remains unclear. Our findings may also indicate that PCOS phenotype D differs substantially from the classic form of the syndrome not only in the severity of metabolic disturbances but also in immunological alterations, despite the presence of low-grade inflammation. The HIF-1α pathway may contribute to the pathogenesis of PCOS by modulating metabolic processes as well as affecting the immune system, including T-cell differentiation, which may significantly modulate the inflammatory response, but the underlying mechanisms warrant further investigation.

A limitation of our study is the relatively small sample size; however, the cited studies assessing the number of Treg and Th17 cells in patients with PCOS were also conducted in small cohorts. Additionally, we studied patients with the D phenotype, without pronounced hyperandrogenism, which may also influence HIF-1α expression as well as the abundance of Treg and Th17 cells. To obtain more valuable findings, it is necessary to consider a larger number of patients and measure the concentrations of Th-related cytokines, especially IFN-γ, TNF-α, interleukin 10 and TGF-β.

Although several correlations reached statistical significance in the initial analysis, these associations did not remain significant after correction for multiple comparisons using the FDR method. Therefore, these findings should be interpreted with caution and require confirmation in larger cohorts.

A major strength of our study is the focus on phenotype D PCOS, a subgroup not previously investigated in this context. To date, all studies concerning the number of Th17 cells and Treg in PCOS have been conducted in patients with classic PCOS phenotypes, characterized by marked hyperandrogenemia and metabolic disturbances. Furthermore, to our knowledge, there are no reports in the literature regarding HIF-1α expression in lymphocytes of patients with PCOS. To date, HIF-1α expression has been evaluated mainly in ovarian granulosa cells, endometrial cells, and ovarian follicular fluid in patients with PCOS.

## 4. Materials and Methods

The study was performed on an ethnically homogenous population of 89 women aged 19–42 years, from the Lower Silesia Region in Poland, comprising 49 women with PCOS and 40 healthy controls comparable in terms of age, weight and BMI. All PCOS patients were selected from cases seen by the Department of Endocrinology and Internal Diseases, University Hospital in Wroclaw, between January 2024 and December 2025.

All women in the PCOS group presented PCOS phenotype D, i.e., oligomenorrhoea and polycystic ovaries on ultrasound without biochemical and/or clinical hyperandrogenism, according to Rotterdam’s criteria [2]. The control group was recruited from healthy volunteers, with regular and ovulatory menstrual cycles.

Exclusion criteria were pregnancy; drug use (such as glucocorticoids, oral contraceptives, statins, fibrates, metformin, and non-steroidal anti-inflammatory drugs); alcohol and tobacco use; chronic, inflammatory or neoplastic disorders; cardiovascular disorders; hypertension; diabetes; hypo- and hyperthyroidism; hypercortisolemia; 21-hydroxylase deficiency; hyperprolactinemia; and virilizing adrenal or ovarian tumor. None of the patients had received recent vaccinations. None of the patients included in the study reported sleep disturbances or strenuous physical activity. Patients had not been on a special diet. The bioethics committee of Wroclaw Medical University approved the protocol of the study (approval decision number: KB192/2023N). All subjects signed informed consent forms in line with the Declaration of Helsinki.

### 4.1. Anthropometric Measurements and Laboratory Assays

Anthropometric measurements were performed on all patients according to the World Health Organization: weight and height, and waist and hip circumference.

Body mass index (BMI) and waist-to-hip ratio (WHR) were calculated using the standard formulas: BMI = body weight (kg)/height (m)^2^; WHR = waist circumference (cm)/hip circumference (cm).

The Homeostatic Model Assessment-Insulin Resistance (HOMA-IR) was calculated by the formula [fasting glucose [nmol/L] × (fasting insulin [mUI/L])/22.5] and the Free Androgen Index (FAI) by the formula testosterone [nmol/L] × 100/sex hormone-binding globulin (SHBG) [nmol/L], according to Vermeulen [85].

Blood samples were drawn from the cubic vein in the morning after an overnight fast, at the follicular phase (between 3rd and 6th day of menstrual cycle), and immediately centrifuged at +4 °C and stored at −80 °C. Each aliquot was thawed only once for analysis.

Biochemical and hormonal parameters included: glucose, total cholesterol (TC), high-density lipoprotein cholesterol (HDL-C), triglycerides (TGs), low-density lipoprotein cholesterol (LDL-C), high sensitive C-reactive protein (hs-CRP) (measured by the enzymatic method using Alinity by Abbott Laboratories, Abbott Park, IL, USA), insulin, luteinizing hormone (LH), follicle-stimulating hormone (FSH), testosterone and sex hormone-binding globulin (SHBG), and 17-hydroxyprogesterone (measured by the chemiluminescent method using Immulite 2000× Pi by Siemens Healthineers, Forchheim, Germany). Interleukin-17 was assessed using the ELISA method (The Diaclone Human Il-17A ELISA kit, Besancon, France).

Ovarian ultrasound was performed on all participants between the 6th and 10th day of the menstrual cycle. The Ferriman–Gallwey score (to evaluate and quantify hirsutism) was used for all participants.

### 4.2. Flow Cytometry

Mouse anti-human monoclonal antibodies were used for analysis: anti-human Fox-P3 AF488 clone 259D (BioLegend, San Diego, CA, USA); anti-human CD25 PerCP-Cy5.5 clone BC96 (BioLegend, San Diego, CA, USA), anti-human CD161 PE-Cy7 clone HP-3G10 (BioLegend, San Diego, CA, USA), anti-human HIF-1α AF647 clone 546-16 (BioLegend, San Diego, CA, USA), anti-human CD4 PB clone OKT4 (BioLegend, San Diego, CA, USA), and anti-human CD3 APC-H7 clone SK7 (Becton Dickinson and Company, San Jose, CA, USA); and anti-human CD45 V500-C clone 2D1 (Becton Dickinson and Company, San Jose, CA, USA) and anti-human ROR gamma (t) PE clone AFKJS-9 (Invitrogen, Carlsbad, CA, USA). A total of 10 mL of blood was collected into EDTA tubes (Sarstedt, Nümbrecht, Germany). The sample was then immediately delivered to the flow cytometry laboratory, no later than 2 h after collection. At the same time, peripheral blood counts were performed. Then human PBMCs (peripheral blood mononuclear cells) were separated using Ficoll–Hypaque (Sigma, St Louis, MO, USA). Cells were surface-stained and conjugated using mouse anti-human monoclonal antibodies in one tube: CD25, CD161, CD4, CD45, and CD3. After incubation, the cells were rinsed, then fixed and permeabilized using a Fix/Perm Buffer Set (BL). The prepared sample was stained intracellularly using HIF-1α, RORγt, and Fox-P3 antibodies.

**Figure 1:**
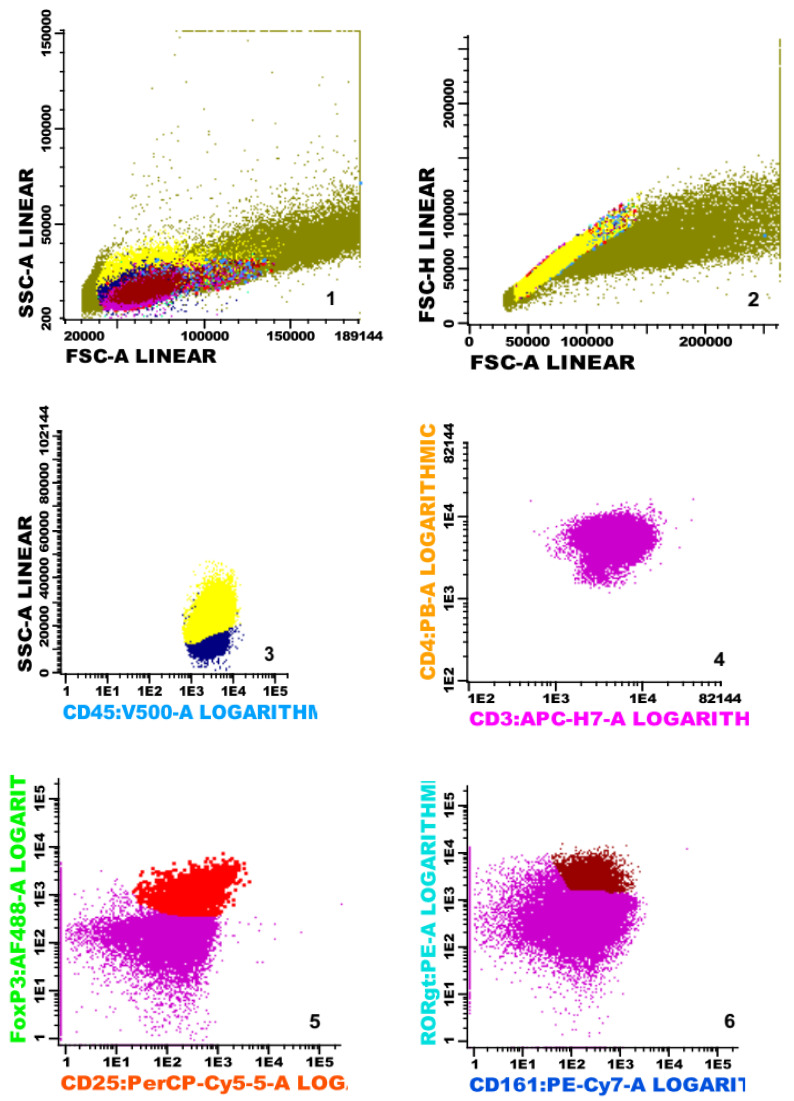

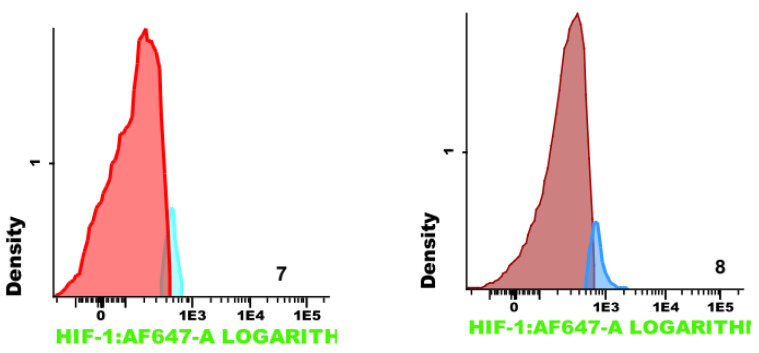
The gating strategy: **1**–**8**: green—debris/doublets, dark blue—lymphocytes, yellow—monocytes, pink—T helper lymphocytes; red—T regulatory lymphocytes; brown—Th17 lymphocytes; light blue—T regulatory HIF-1α-positive lymphocytes, blue—Th17 HIF-1α positive lymphocytes. **1**—discrimination of debris (FSC-A vs. SSC-A); **2**—discrimination of debris and doublets (FSC-A vs. FSC-H); **3**—lymphocyte and monocyte gating (CD45 vs. SSC-A); **4**—T helper lymphocyte gating (CD3 vs. CD4); **5**—T regulatory lymphocytes (CD25 vs. FoxP3); **6**—Th17 lymphocytes (CD161 vs. RORγt); **7**—T regulatory HIF-1α-positive lymphocytes; (CD25 vs. FoxP3); **8**—Th17 HIF-1α-positive lymphocytes.

The evaluation of nucleated cells was carried out on an 8-color FACSCanto II flow cytometer (BD). In each test tube, we collected as many cells as possible. On average, there were 3 × 10^5^ cells. The data were analyzed using BD Infinicyt software v 2.0; the gating strategy is shown in Figure 1. Regulatory T cells (Tregs) were defined as CD45+,CD3+, CD4+, CD25high+ and Fox-P3+ cells. Th17 T cells were defined as CD45+, CD3+, CD4+, CD161+ and RORγt+ cells. The FMO (fluorescence minus one) strategy was used as a control for the experiment.

### 4.3. Statistical Analysis

Data were analyzed using Statistica software for Windows (version 13.3, StatSoft, Krakow, Poland). Results are presented as mean with standard deviation (SD), median, and interquartile range (IQR). The Shapiro–Wilk test was used to assess data distribution. Student’s *t*-test or the Mann–Whitney test was applied to compare quantitative variables. The homogeneity of variances was verified by Levene’s test. Correlations between parameters were calculated using Pearson’s test or Spearman’s rank correlation test. A *p*-value less than 0.05 was considered statistically significant. The power of the Mann–Whitney test was calculated using a bootstrap procedure. For each group, 10,000 resampled datasets of the same size as the original were drawn with replacements, and the Mann–Whitney test was applied to each pair. The proportion of tests with *p* < 0.05 was taken as the calculated power.

## Figures and Tables

**Table 1:** General characteristics of the women with polycystic ovary syndrome and the control group.

Group	PCOS (*n* = 49)			CG (*n* = 40)			*p*-Value
	Mean ± SD	Median	IQR	Mean ± SD	Median	IQR	
Age [years]	27.0 ± 5.3	26.0	23.0–30.0	29.0 ± 5.8	29.0	24.5–34.5	0.083
Body weight [kg]	72.96 ± 21.4	66.0	58.0–83.0	65.0 ± 15.9	59.5	55.0–69.0	0.065
BMI [kg/m^2^]	26.1 ± 7.6	23.6	20.7–29.8	23.7 ± 5.8	21	20.3–24.7	0.100
Waist circumference [cm]	84.4 ± 19.2	74.0	69.0–99.0	77.0 ± 15.4	70.0	69.0–81.0	0.034
Hip circumference [cm]	100.8 ± 15.2	97.0	90.0–110.0	94.4 ± 12.9	90.0	86.0–100.0	0.016
WHR	0.83 ± 0.1	0.80	0.76–0.92	0.81 ± 0.06	0.80	0.76–0.85	0.717

PCOS—polycystic ovary syndrome; CG—control group; BMI—body mass index; WHR—waist-to-hip ratio; SD—standard deviation; IQR—interquartile range.

**Table 2:** Hormonal and biochemical profile in women with polycystic ovary syndrome and the control group.

Group	PCOS (*n* = 49)			CG (*n* = 40)			*p*-Value
	Mean ± SD	Median	IQR	Mean ± SD	Median	IQR	
Testosterone [ng/mL; 0.2–0.8]	0.37 ± 0.1	0.36	0.3–0.4	0.29 ± 0.1	0.27	0.2–0.4	0.006
SHBG [nmol/L; 18–144]	51.2 ± 33.1	41.2	27.0–69.7	56.3 ± 22.2	54.45	36.2–69.1	0.089
FAI [0.55–12]	3.4 ± 2.4	2.5	1.6–4.4	2.2 ± 1.5	1.7	1.2–2.3	0.002
DHEA-S [µg/dL; 35–430]	300.9 ± 134.4	272.0	221.0–398.0	252.7 ± 113.8	240.5	174.5–336.5	0.081
LH [mIU/mL; 1.1–11.6]	8.7 ± 5.1	7.2	5.2–10.8	6.3 ± 3.1	5.6	4.3–8.7	0.038
hsCRP [mg/L; 0.2–5]	3.9 ± 8.7	1.0	0.3–3.0	1.4 ± 2.0	0.65	0.5–0.95	0.046
IL-17 [pg/mL]	33.6 ± 9.2	32.5	27.8–40.1	37.5 ± 10.0	35.5	30.8–40.4	0.061
Fasting glycemia [mg/dL; 70–99]	85.6 ± 7.5	85.6	80.0–92.0	86.0 ± 12.9	83.5	80.0–90.0	0.523
Insulin [µIU/mL; <24]	11.4 ± 16.7	7.1	3.8–10.7	6.8 ± 6.2	4.3	2.1–7.9	0.523
HOMA-IR	2.4 ± 3.5	1.5	0.8–2.5	1.4 ± 1.4	0.8	0.5–1.6	0.059
Total cholesterol [mg/dL; <190]	185.8 ± 37.1	178.5	156.5–207.5	180.0 ± 32.7	182.0	157.0–208.0	0.658
HDL cholesterol [mg/dL; >40]	63.8 ± 15.0	63.0	54.0–75.0	64.0 ± 15.4	63.0	56.0–69.0	0.842
LDL cholesterol [mg/dL; <135]	104.0 ± 34.0	98.0	81.0–118.5	102.6 ± 26.2	102.0	85.0–125.0	0.763
Triglycerides [mg/dL; <160]	88.0 ± 70.5	66.0	51.0–94.0	66.2 ± 33.0	57.0	46.0–73.0	0.127

PCOS—polycystic ovary syndrome; CG—control group; SD—standard deviation; SHBG—sex hormone-binding globulin; FAI—free androgen index; DHEA-S—dehydroepiandrosterone sulfate; LH—luteinizing hormone; hsCRP—high sensitive C-reactive protein; IL-17—interleukin 17; HDL cholesterol—high-density lipoprotein cholesterol; LDL cholesterol—low-density lipoprotein cholesterol; IQR—interquartile range.

**Table 3:** Peripheral blood count parameters in women with polycystic ovary syndrome and the control group.

Group	PCOS (*n* = 49)			CG (*n* = 40)			*p*-Value
	Mean ± SD	Median	IQR	Mean ± SD	Median	IQR	
Leukocytes [10^3^/µL]	6.6 ± 2.6	5.9	5.0–7.2	5.7 ± 1.8	5.5	4.-6.4	0.163
Lymphocytes [10^3^/µL]	1788.5 ± 597.8	1714.2	1375.2–2038.8	1630.9 ± 414.1	1595.1	1301.0–1909.4	0.332
T lymphocytes [µl]	1234.6 ± 453.4	1177.8	900.7–1501.3	1210.4 ± 334.3	1193.5	939.3–1456.8	0.948
T CD4+ lymphocytes [µl]	704.8 ± 113.0	610.0	510.5–839.4	694.7 ± 214.0	669.0	523.1–842.0	0.551
Tregs [µl]	47.9 ± 25.4	43.2	30.8–58.1	40.2 ± 14.6	39.6	29.3–51.3	0.345
Th17 [µl]	10.9 ± 7.3	9.8	6.5–13.7	12.7 ± 7.0	10.6	6.9–16.8	0.231
Tregs HIF-1α + [µl]	4.6 ± 9.1	1.8	0.8–5.4	1.9 ± 2.1	1.3	0.4–3.0	0.107
Th17 HIF-1α + [µl]	1.6 ± 1.7	1.2	0.6–1.9	1.4 ± 1.1	1.0	0.4–2.3	0.692
Tregs/Th17	6.8 ± 10.3	4.8	3.0–7.1	3.7 ± 1.6	3.4	2.6–4.3	0.009
Tregs HIF-1α+/TH17 HIF-1α +	3.1 ± 5.7	1.6	1.1–2.9	1.4 ± 0.7	1.4	0.9–1.8	0.038
T CD3+ lymphocytes HIF-1α + [µl]	31.6 ± 32.2	20.9	7.2–46.8	23.5 ± 24.9	11.0	5.1–36.9	0.256
T helper CD4+ lymphocytes HIF-1α + [µl]	17.5 ± 19.8	11.2	4.2–25.6	13.1 ± 13.9	6.9	3.0–21.9	0.275

PCOS—polycystic ovary syndrome; CG—control group; Tregs—regulatory T cells; Th17—T helper 17 cells; HIF-1α—hypoxia-inducible factor 1α; SD—standard deviation; IQR—interquartile range.

## Data Availability

The original contributions presented in the study are included in the article; further inquiries can be directed to the corresponding authors.
